# Enzymatic measurement of phosphatidylglycerol and cardiolipin in cultured cells and mitochondria

**DOI:** 10.1038/srep11737

**Published:** 2015-06-30

**Authors:** Shin-ya Morita, Tomohiro Terada

**Affiliations:** 1 Department of Pharmacy, Shiga University of Medical Science Hospital, Otsu City, Shiga 520-2192, Japan

## Abstract

Phosphatidylglycerol (PG) and cardiolipin (CL) are synthesized in mitochondria and regulate numerous biological functions. In this study, a novel fluorometric method was developed for measuring PG and CL using combinations of specific enzymes and Amplex Red. This assay quantified the sum of PG and CL (PG + CL) regardless of the species of fatty acyl chain. The calibration curve for PG + CL measurement was linear, and the detection limit was 1 μM (10 pmol in the reaction mixture). This new method was applied to the determinations of PG + CL content in HEK293 cells and CL content in purified mitochondria, because the mitochondrial content of PG is negligible compared with that of CL. We demonstrated that the PG+CL content was greater at low cell density than at high cell density. The overexpression of phosphatidylglycerophosphate synthase 1 (PGS1) increased the cellular contents of PG + CL and phosphatidylcholine (PC), and reduced that of phosphatidic acid. PGS1 overexpression also elevated the mitochondrial contents of CL and PC, but had no effect on the number of mitochondria per cell. In addition to the enzymatic measurements of other phospholipids, this simple, sensitive and high-throughput assay for measuring PG + CL can be used to understand cellular, physiological and pathological processes.

The anionic phospholipids phosphatidylglycerol (PG) and cardiolipin (CL) are important for cellular functions in all eukaryotes and some prokaryotes. In mammals, PG is a minor phospholipid component of many intracellular membranes, accounting for less than 1% of total phospholipids, and is located mostly in the mitochondrial and microsomal membranes^1^,^2^. In lung, PG is one of the main components of lung surfactant and localized predominantly in lamella body membranes. PG content in rabbit sperm is relatively high, being 6.8% of total phospholipids^3^. In cyanobacteria and chloroplasts of higher plants, the majority of PG is found in thylakoid membranes, which are the site of photosynthetic light reactions and electron transport^4^. On the other hand, CL constitutes 0.2–15% of total phospholipids in various mammalian tissues and is present at its highest concentrations in cardiac muscles. Most of the CL molecules in cells are associated with the inner mitochondrial membranes, whereas only trace amounts of CL are detected in the outer mitochondrial membranes^1^,^2^,^5^,^6^,^7^.

In mammalian cells, PG is produced from CDP-diacylglycerol (CDP-DAG) through two steps catalyzed by phosphatidylglycerophosphate (PGP) synthase and PGP phosphatase. CDP-DAG is formed from phosphatidic acid (PA) by the enzymes CDP-DAG synthase 1 and 2, which are integral membrane proteins located to mitochondria and endoplasmic reticulum^2^,^6^,^8^. CDP-DAG is converted to PGP by the action of the mitochondrial enzyme PGP synthase 1 (PGS1) through the exchange of glycerol-3-phosphate (G3P) with CMP moiety of CDP-DAG^2^,^6^,^9^,^10^. PGP synthase activity is abundant in the inner mitochondrial membrane. The highest expression level of *PGS1* mRNA is found in the testis, whereas the level of the *PGS1* mRNA in lung containing a high level of PG is similar to those in other tissues such as skeletal muscle and liver^9^. PGP is rapidly dephosphorylated to generate PG. PTPMT1 identified as a PGP phosphatase is anchored to the matrix side of the inner mitochondrial membrane^7^,^11^,^12^. CL is synthesized by the condensation of PG and CDP-DAG at the mitochondrial inner membrane, which is catalyzed by CL synthase 1^6^,^13^.

Besides their function in cell membrane homeostasis, PG and CL are mediators of molecular signaling for numerous cellular processes. PG is a potential activator of the protein kinase C family^14^,^15^. CL stabilizes the electron transfer complex in the inner mitochondrial membranes and promotes the production of ATP as a structural component of the ATP/ADP carrier and respiratory complexes III and IV^16^. Complete absence of CL in yeast mitochondria results in partially defective protein import into mitochondria and decreased mitochondrial membrane potential^17^. CL also serves as a central switch in the mitochondrial apoptotic program and is directly involved in mitochondrial outer membrane permeabilization by enabling docking and activation of pro-apoptotic Bcl-2 proteins^6^,^18^,^19^. CL is closely associated with cytochrome *c* at the outer leaflet of the mitochondrial inner membrane, and CL peroxidation is critical for cytochrome *c* dissociation from the mitochondrial inner membrane^6^,^18^,^19^,^20^. Although alterations in the abundance and molecular form of CL are associated with pathological states, including aging, ischemia and reperfusion, heart failure, inherited and diabetic cardiomyopathy, and cancer^7^,^21^,^22^,^23^,^24^,^25^, detailed information about their molecular role remains unknown. A genetic high density lipoprotein deficiency, Tangier disease, is also linked to CL abnormalities^26^. A severe genetic disorder, Barth syndrome, is associated with impaired CL acyl-chain remodeling through mutations in Tafazzin, and Barth syndrome patients suffer from cyclic neutropenia, and skeletal and cardiac myopathies^2^,^7^. Although the mitochondrial CL is normally hidden from the immune system, anti-CL antibodies are present in patients suffering from anti-phospholipid syndrome^7^. It is still unclear whether the antibodies are generated against mitochondrial or bacterial CL.

Therefore, quantifications of PG and CL are required to understand the cellular, physiological and pathological mechanisms, and several methods have been developed. The conventional assay for measuring PG and CL includes separation by thin-layer chromatography followed by quantification of phosphate from the spot, which has low sensitivity and low throughput. Mass spectrometry (MS) has emerged as a method for the characterization and quantification of PG and CL molecular species that differ in acyl-chain composition^6^,^27^. However, it is difficult to determine the total concentration of PG or CL using mass spectrometry. The immunological agglutination test for the detection of PG is rapid and simple, but insensitive^28^. To measure PG and CL newly synthesized in response to stimuli, cellular phospholipids are metabolically labeled with radioisotopes such as ^32^P, and then radioactive PG and CL are quantified following lipid extraction and separation by thin-layer chromatography^9^, which is time-consuming and has low throughput. It is an important issue whether the measurement of labeled phospholipid is a true reflection of the unlabeled phospholipid, because the labeling extent may depend on many factors, including metabolism, localization, pooling, and equilibration time, and may be different among phospholipid classes. There is no sensitive and simple method for PG and CL measurement. We have developed the enzymatic fluorometric assays for quantifications of PA, phosphatidylcholine (PC), phosphatidylethanolamine, phosphatidylserine and sphingomyelin^29^,^30^,^31^,^32^. In this study, we aimed to develop an enzyme-based fluorometric method for quantifying the sum of PG and CL (PG + CL), which can be used routinely to measure PG + CL levels in cells and mitochondria. This new assay can provide simple, sensitive and high-throughput quantification of PG + CL. Using this novel method, we also investigated the relationship between cell density and PG + CL content and the effect of PGS1 overexpression on the PG + CL content in cells and mitochondria.

## Results and Discussion

### PG + CL measurement

**Figure 1:**
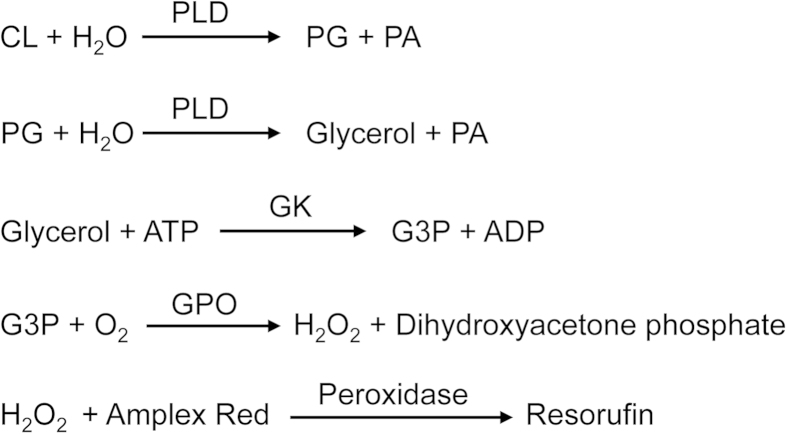
Strategy for PG + CL measurement. PLD catalyzes the hydrolysis of CL to PG and PA and the subsequent hydrolysis of PG to glycerol and PA. Glycerol is phosphorylated by glycerol kinase to G3P. Oxidation of G3P is catalyzed by GPO, which produces hydrogen peroxide. In the presence of peroxidase, Amplex Red reacts with hydrogen peroxide to produce highly fluorescent resorufin, which can be measured.

Phospholipase D (PLD) hydrolyzing CL to release glycerol has not been identified. Using PLD from *Streptomyces chromofuscus*, we developed a new method for the enzymatic measurement of PG + CL, which involves five steps (Fig. 1). (1) PLD hydrolyzes CL to PG and PA. (2) PG is subsequently hydrolyzed to glycerol and PA by PLD. (3) Glycerol kinase (GK) catalyzes the phosphorylation of glycerol to form G3P. (4) G3P is oxidized by G3P oxidase (GPO), which generates hydrogen peroxide and dihydroxyacetone phosphate. (5) Finally, in the presence of peroxidase, hydrogen peroxide reacts with Amplex Red to generate highly fluorescent resorufin, which can be measured. This method requires a sample volume of only 10 μl in a 96-well format.

**Figure 2:**
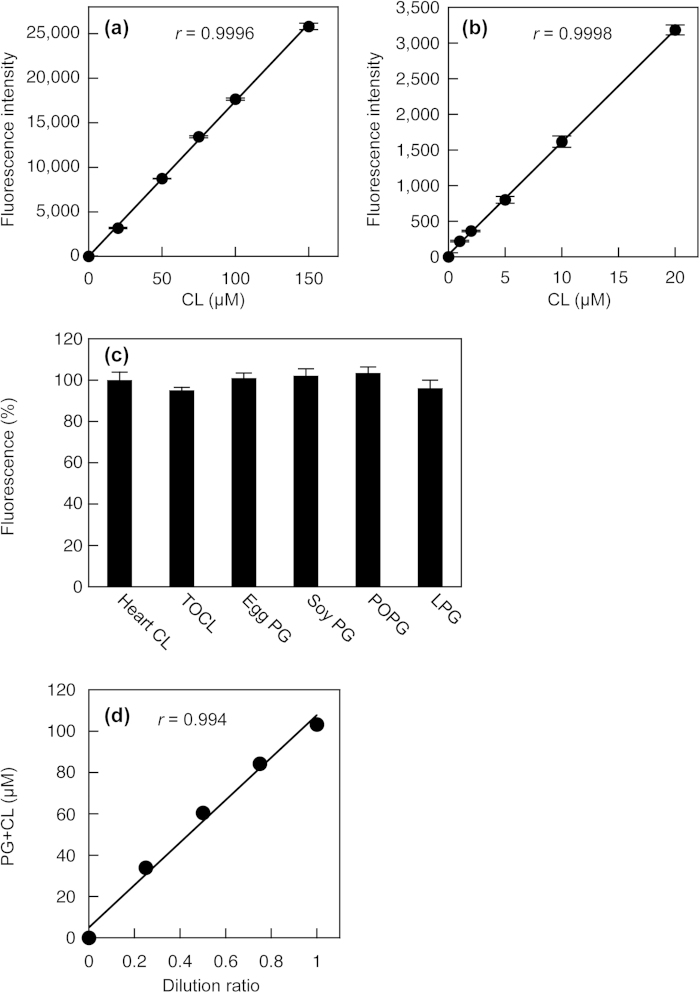
Enzymatic measurement of PG + CL. (**a**) and (**b**) Standard curves for PG + CL measurement. The heart CL standard solution was added to Reagent L1 and incubated at 37 °C for 30 min. Then, Reagent L2 was added. After 30 min of incubation at room temperature, Stop Reagent was added. The fluorescence intensity was measured using a microplate reader. Background fluorescence was 3973 ± 57, which was subtracted from each value. Each point represents the mean ± S.D. of triplicate measurement. The lines were obtained by linear regression analysis. The correlation coefficients were *r* = 0.9996 (**a**) and *r* = 0.9998 (**b**). (**c**) Fluorescence changes in response to heart CL, TOCL, egg PG, soy PG, POPG and LPG in PG + CL measurement. Each bar represents the mean ± S.D. of triplicate measurement. There were no statistically significant differences between heart CL, TOCL, egg PG, soy PG, POPG and LPG. (**d**) Linearity of PG + CL measurement. The lipid extract from HEK293 cells was sequentially diluted with 1% Triton X-100. The correlation coefficient was *r* = 0.994.

A calibration reaction was performed using heart CL standard solutions to validate this novel method for PG + CL measurement. As shown in Fig. 2a,b, there was a linear relationship to 150 μM CL (*r* = 0.9996), and the detection limit was as low as 1 μM (10 pmol in the reaction mixture). CL, PG and lysophosphatidylglycerol (LPG) increased the fluorescence to the same extent when normalized to the moles (Fig. 2c), indicating that PLD from *Streptomyces chromofuscus* can hydrolyze CL, PG and LPG to release glycerol. Hence, this measurement cannot distinguish between CL, PG and LPG. There was no significant difference in the fluorescence change in response to heart CL containing mixed acyl chains and tetraoleoyl CL (TOCL). In addition, the fluorescence change in response to L-α-palmitoyl-oleoyl PG (POPG) was equal to that in response to egg PG or soy PG containing mixed acyl chains. Therefore, the chain length or the number of double bonds does not affect this PG + CL measurement.

### Measurement of PG + CL in cultured cells

Cultured cells contain a substantial amount of G3P, which reacts with GPO and confuses the PG + CL measurement. The assay is also confounded by H_2_O_2_, which can be removed by lipid extraction^33^. The method of Bligh and Dyer has been widely used for lipid extraction, followed by quantifications of PG and CL using radiolabeled phosphate, TLC or mass spectrometry^9^,^23^,^25^. In the method of Bligh and Dyer using chloroform, methanol and water (see Supplementary Methods), H_2_O_2_ and G3P partition into the upper aqueous phase, whereas lipids partition into the lower organic phase. We examined how effectively H_2_O_2_ and G3P can be removed from samples by the Bligh and Dyer lipid extraction. The enzymatic assays have been generally used to determine the concentrations of H_2_O_2_ and G3P^34^,^35^,^36^. The calibration curves for enzymatic fluorometric measurements of H_2_O_2_ and G3P were linear (Supplementary Figures S1 and S2). Although the sample solutions contained 10–1,000 μM H_2_O_2_ before the lipid extraction, the samples after the lipid extraction contained no detectable concentration of H_2_O_2_ (<1 μM) (Supplementary Figure S1). In aerobic cells, the level of H_2_O_2_ is regulated at 0.001–0.1 μM depending on the H_2_O_2_ production^37^. At sites of inflammation, H_2_O_2_ generated by activated phagocytes modulates the inflammatory process^38^. Substantial quantities of H_2_O_2_ (>20 μM) are sometimes detected in freshly voided urine^38^. High levels of H_2_O_2_ (≥50 μM) are cytotoxic to a wide range of animal, plant and bacterial cells in culture^38^. We demonstrated that the lipid extraction by the method of Bligh and Dyer can completely remove the extremely high concentration (1,000 μM) of H_2_O_2_. As shown in Supplementary Figure S2, no detectable concentration of G3P (<1 μM) remained in the samples after the lipid extraction, even though the samples before the lipid extraction contained 500 μM G3P, which is much higher concentration compared with various cellular samples^35^,^39^. We also confirmed that the lipid extract from HEK293 cells did not contain detectable amount of H_2_O_2_ or G3P (<1 μM), indicating intrinsic H_2_O_2_ and G3P of HEK293 cells were completely removed by the Bligh and Dyer lipid extraction. Additionally, we have previously demonstrated that the lipid extract from rat astrocytes, even though treated with 100 μM H_2_O_2_, did not contain detectable amount of H_2_O_2_ or G3P^33^. Thus, to remove the contaminating G3P and H_2_O_2_, lipid extraction from cells is recommended for the enzymatic measurement of PG + CL. The lipid extraction is widely used before the enzymatic assays for quantifying phospholipids, cholesterol and triglyceride in cells or animal tissues, which also measure H_2_O_2_ at the final step^40^. In general, the content of LPG in mammalian cells is extremely low compared to the contents of PG and CL, and negligible for the PG + CL measurement.

To assess the accuracy of the method, we performed a recovery test, in which known quantities of heart CL were added to the lipid extract from HEK293 cells (Table 1). The mean recovery of CL in concentrations of 12.5–100 μM was 100.2%, indicating no interference of hydrophobic compounds extracted from the cells.

To test the linearity of the measurement, the cellular lipid extract was sequentially diluted with 1% Triton X-100 aqueous solutions. Figure 2d shows a well-fitted regression line to 103 μM PG + CL (*r* = 0.994).

PG is a common precursor for CL and bismonoacylglycerophosphate (BMP)^6^. BMP is a structural isomer of PG, and each glycerol moiety of BMP is esterified through the *sn*-1 position to the phosphate and contains a single fatty acid ester^41^. The lysosomal PLA2G15 or the methyl-arachydonyl fluorophosphonate-sensitive cytoplasmic PLA_2_ may be involved in the conversion of PG to BMP^6^. PLA2G15 has a Ca^2+^-independent PLA_2_ and transacylase activity. BMP is preferentially located in lysosomes and endosomes, and also present in exosomes^6^,^41^,^42^. On the other hand, PC is also a precursor of CL synthesis, because mammalian PLD1 and PLD2 mainly catalyze the hydrolysis of PC to PA^2^. PLD1 is present primarily in a perinuclear region of the Golgi apparatus, late endosomes, and endoplasmic reticulum, while the major location of PLD2 is the plasma membrane^2^. The presented enzymatic method measures the sum of PG and CL, although the metabolic pathways of phospholipids including PG and CL are very complicated.

The shotgun approach based on MS/MS is frequently applied for CL identification and quantification^25^,^43^,^44^,^45^. For quantification, MS analysis is conducted in negative ionization mode. Shotgun or direct infusion MS offers advantages in untargeted analysis and coverage of a broad spectrum of CL species. Identification and quantification of CL molecular species are performed by using a precursor ion scan on the most abundant linoleate fatty acid, but the analysis of minor and overlapping CL molecular species is difficult. The two-dimensional MS technique takes into account the CL building blocks, including glycerophosphate, the neutral loss of ketenes and all of the potential naturally occurring fatty acyl carboxylates and enables the identification and quantification of complex CL structures down to the fatty acid level^44^. On the other hand, MS with liquid chromatography offers advantages in sensitivity and selectivity. Sensitivity down to the femto-molar range is accomplished by targeted analysis using liquid chromatography–MS/MS in the selected reaction monitoring-mode^46^,^47^,^48^. The analysis of CL species is performed with normal phase chromatography^46^,^47^,^49^. BMP and PG are structural isomers with a large number of molecular species. However, Scherer *et al.* have reported the simultaneous quantification of PA, PG, CL and BMP species involved in the polyglycerophospholipid pathways using hydrophilic interaction liquid chromatography-MS/MS in positive electrospray ionization mode^49^. The total run time of this analysis is 6–8 min. However, this method takes a long time to analyze many samples compared with the enzymatic assay. These MS methods do not quantify the total amount of PG or CL, and the obtained data are different between mass spectrometry and enzymatic assay. Because we have developed the enzymatic fluorometric assays of PC, phosphatidylethanolamine, phosphatidylserine, PA and sphingomyelin, this enzymatic assay of PG + CL is useful to assess the compositional profile of phospholipids in various membranes, in addition to MS analysis.

### Effect of cell density on PG + CL content in HEK293 cells

**Figure 3:**
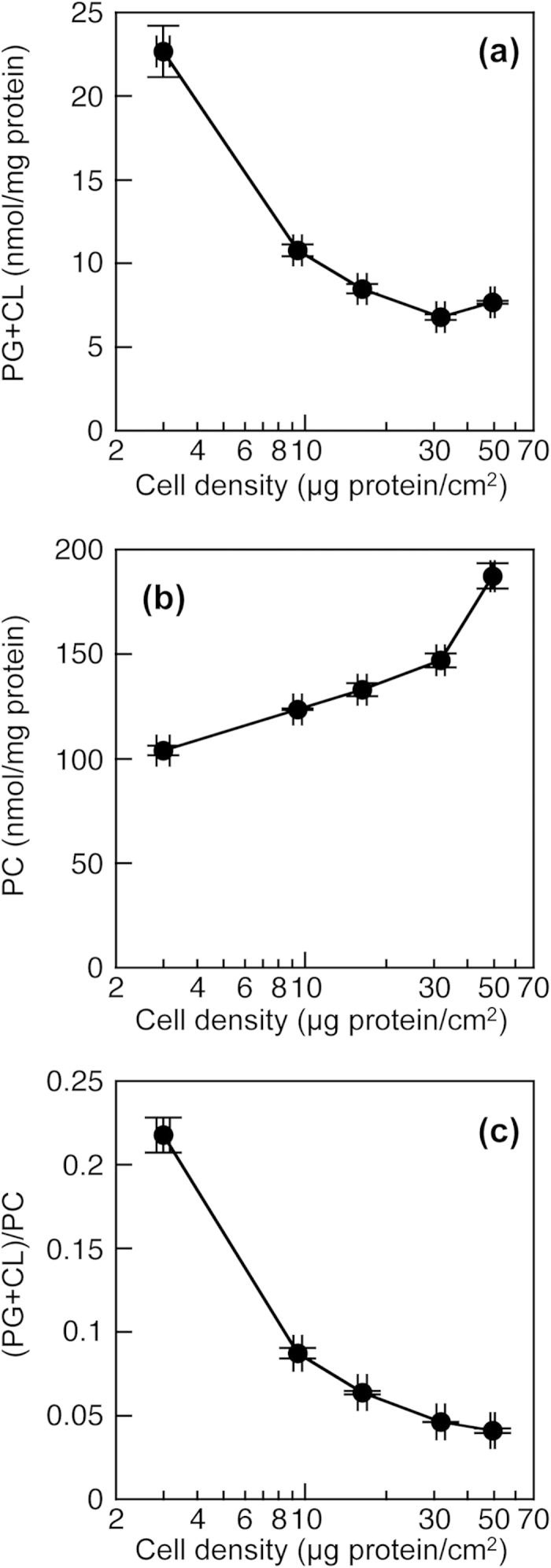
Effect of cell density on PG + CL content in HEK293 cells. HEK293 cells on 10 cm dishes were incubated in MEM containing 0.02% BSA for 18 h at 37 °C. PG + CL content (**a**), PC content (**b**) and (PG + CL)/PC ratio (**c**) of HEK293 cells were determined by the enzymatic measurements and protein assay. Each point represents the mean ± S.E. of three measurements.

Although various cellular processes are influenced by cell density, the relationships between cell density and contents of PG and CL have not been evaluated because of the difficulty in determining the contents of PG and CL in sparse cell cultures by previous methods. Using the new enzymatic measurement of PG + CL, we investigated the effect of cell density on the PG + CL content in HEK293 cells. The cellular PG + CL content decreased with increasing cell density and became constant at higher cell densities (Fig. 3a). On the other hand, the cellular PC content increased in a cell density-dependent manner (Fig. 3b), which was in accordance with our previous results^29^,^30^,^32^. We also determined the (PG + CL)/PC ratio at various cell densities. The (PG + CL)/PC ratio is an index of the PG + CL concentration in the cell membrane because PC is the most abundant lipid in the mammalian cell membranes. As a result, the (PG + CL)/PC ratio was negatively correlated with the cell density (Fig. 3c). We have previously observed that an increase in the density of HEK293 cells is accompanied by elevations in the cellular contents of PC, PE and SM, but by decreases in the PA and PS contents^29^,^30^,^31^,^32^. Collectively, these findings suggest that the compositions of all membrane phospholipids including PG and CL are regulated by the signaling from cell-cell adhesion and/or cellular maturation.

### Effect of PGS1 overexpression on PG + CL content in HEK293 cells

**Figure 4:**
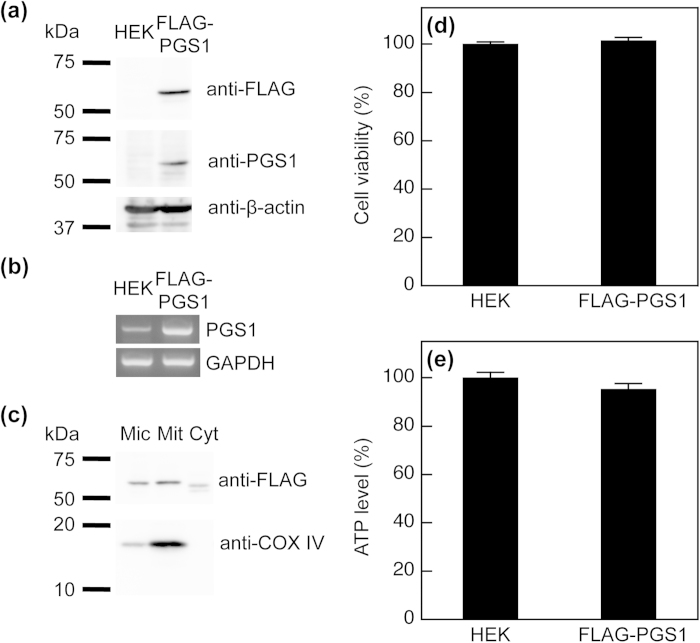
Expression of FLAG-PGS1 in HEK293 cells. (**a**) Immunoblot analysis of FLAG-PGS1. Sonically disrupted cell lysates (19.1 μg of protein) from HEK293 and HEK/FLAG-PGS1 cells were separated by 10% SDS-PAGE. The N-terminus of PGS1 was fused to the FLAG-tag, and FLAG-PGS1 was detected with an anti-FLAG antibody or with a polyclonal anti-PGS1 antibody (immunogen aa 110-556). (**b**) Expression of PGS1 mRNA in HEK293 and HEK/FLAG-PGS1 cells was assessed by RT-PCR. (**c**) Microsomal (Mic), purified mitochondrial (Mit) and cytosolic (Cyt) fractions were isolated from HEK/FLAG-PGS1 cells. Proteins (39.5 μg) were separated by 10% SDS-PAGE and then immunoblotted with an anti-FLAG antibody. COX IV (mitochondrial marker) was detected with a specific antibody. (**d**) and (**e**) Cell viability and intracellular ATP level were assessed by the resazurin assay and the bioluminescence-based assay, respectively. Data were reported as the percentages of controls. Each bar represents the mean ± S.E. of four measurements. There were no significant differences in cell viability and ATP levels between HEK293 and HEK/FLAG-PGS1 cells.

For PG and CL synthesis, PGS1, a 556-amino-acid enzyme, catalyzes the formation of PGP from CDP-DAG and G3P^2^,^6^. Subsequently, PGP phosphatase dephosphorylates PGP to PG, which is converted to CL by reacting with CDP-DAG under the control of CL synthase^2^,^6^. To ascertain whether the alteration in cellular PG + CL content can be detected using our new method, we constructed a HEK293 cell line stably expressing FLAG-PGS1 (HEK/FLAG-PGS1). A FLAG-tag was fused to the N-terminus of PGS1 for immunodetection using anti-FLAG antibody. FLAG-PGS1 was expressed as a protein migrating at ~60 kDa on SDS/PAGE (Fig. 4a). The expression of FLAG-PGS1 in the stable cell line was also detected using polyclonal anti-PGS1 antibody, but endogenous PGS1 was not detected in the host HEK293 cells. Although PGS1 mRNA was detected endogenously in HEK293 cells, the expression level of PGS1 mRNA was much higher in HEK/FLAG-PGS1 cells than in the host HEK293 cells (Fig. 4b). Kawasaki *et al.* have shown that the PGS1 protein is abundant in mitochondria of CHO cells^10^. To determine whether FLAG-PGS1 protein was localized to mitochondria, we isolated purified mitochondrial, microsomal and cytosolic fractions from HEK/FLAG-PGS1 cells. Immunoblotting with anti-FLAG antibody showed that FLAG-PGS1 was primarily distributed in the purified mitochondrial fraction (Fig. 4c). The double band migrating faster in the cytosolic fraction was likely to represent degradation products of the FLAG-PGS1 protein, which has also been shown previously^10^. COX IV, a specific marker protein for mitochondria, was recovered predominantly in the purified mitochondrial fraction. These immunoblotting data demonstrate that FLAG-PGS1 resides in mitochondria.

We evaluated the cell viability and intracellular ATP level as a measure of cellular function to exclude the possibility of detrimental effects of the transfection and selection with G418. The cell viability and intracellular ATP levels were not significantly different between HEK293 and HEK/FLAG-PGS1 cells (Fig. 4d,e).

**Figure 5:**
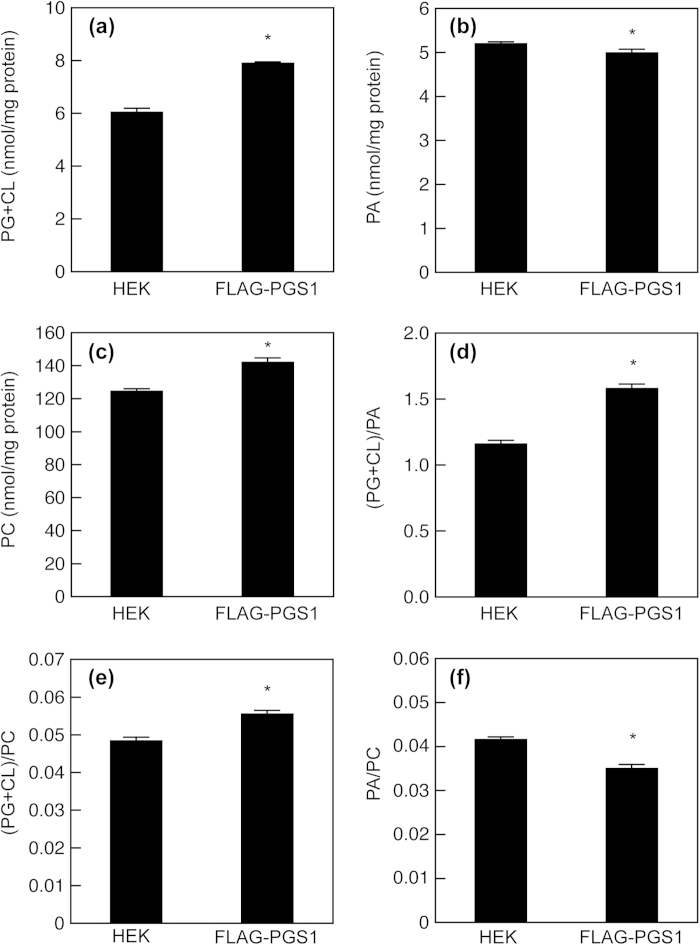
Effect of PGS1 overexpression on membrane phospholipid composition. HEK293 and HEK/FLAG-PGS1 cells on 10 cm dishes were incubated in MEM containing 0.02% BSA for 18 h at 37 °C. There was no difference in the densities of HEK293 and HEK/FLAG-PGS1 cells (39.8 ± 0.5 and 42.2 ± 0.9 μg protein/cm^2^, respectively). PG + CL content (**a**), PA content (**b**), PC content (**c**), (PG + CL)/PA ratio (**d**), (PG + CL)/PC ratio (e) and PA/PC ratio (**f**) of the cells were determined by enzymatic measurements of PG + CL, PA and PC, and protein assay. Each bar represents the mean ± S.E. of four measurements. *P < 0.05, significantly different from HEK293 cells.

To examine the effect of PGS1 overexpression on cellular phospholipid metabolism, we quantified the contents of PG + CL, PC and PA in HEK293 and HEK/FLAG-PGS1 cells at similar cell densities (39.8 ± 0.5 and 42.2 ± 0.9 μg protein/cm^2^, respectively) by using enzymatic assays. As shown in Fig. 5a, the expression of FLAG-PGS1 resulted in a 31% increase in the PG + CL content. HEK293 cells expressed no detectable PGS1 protein but contained substantial amounts of PG and CL, suggesting that other unknown pathways exist for the PG production in HEK293 cells. The PG + CL content in HEK/FLAG-PGS1 cells was also significantly higher than that in the mock-transfected cells (Supplementary Figure S3). In contrast, the cellular content of PA, a precursor of PG, was slightly but significantly decreased by FLAG-PGS1 expression (Fig. 5b), and thus the (PG + CL)/PA ratio in HEK/FLAG-PGS1 cells was 1.4-fold higher than that in HEK293 cells (Fig. 5d). The overexpression of PGS1 had relatively little effect on the PA content, suggesting that the loss of PA is compensated for by diacylglycerol kinase, phospholipase D or de novo G3P acylation. Unexpectedly, PC was significantly more abundant in HEK/FLAG-PGS1 cells than in HEK293 cells (Fig. 5c). Consequently, HEK/FLAG-PGS1 cells exhibited a higher (PG + CL)/PC ratio and a lower PA/PC ratio than HEK293 cells (Fig. 5e,f). The activity of CTP:phosphocholine cytidylyltransferase, a rate-limiting enzyme in PC synthesis, is regulated by reversible binding to cell membrane lipids. PG and CL generate a negative electrostatic surface potential, which promotes the membrane binding and activation of this enzyme^50^,^51^. Therefore, the enhanced PC production in HEK/FLAG-PGS1 cells may be partially attributed to the activation of CTP:phosphocholine cytidylyltransferase by PG and CL.

### Effect of PGS1 overexpression on CL content in mitochondria

**Figure 6:**
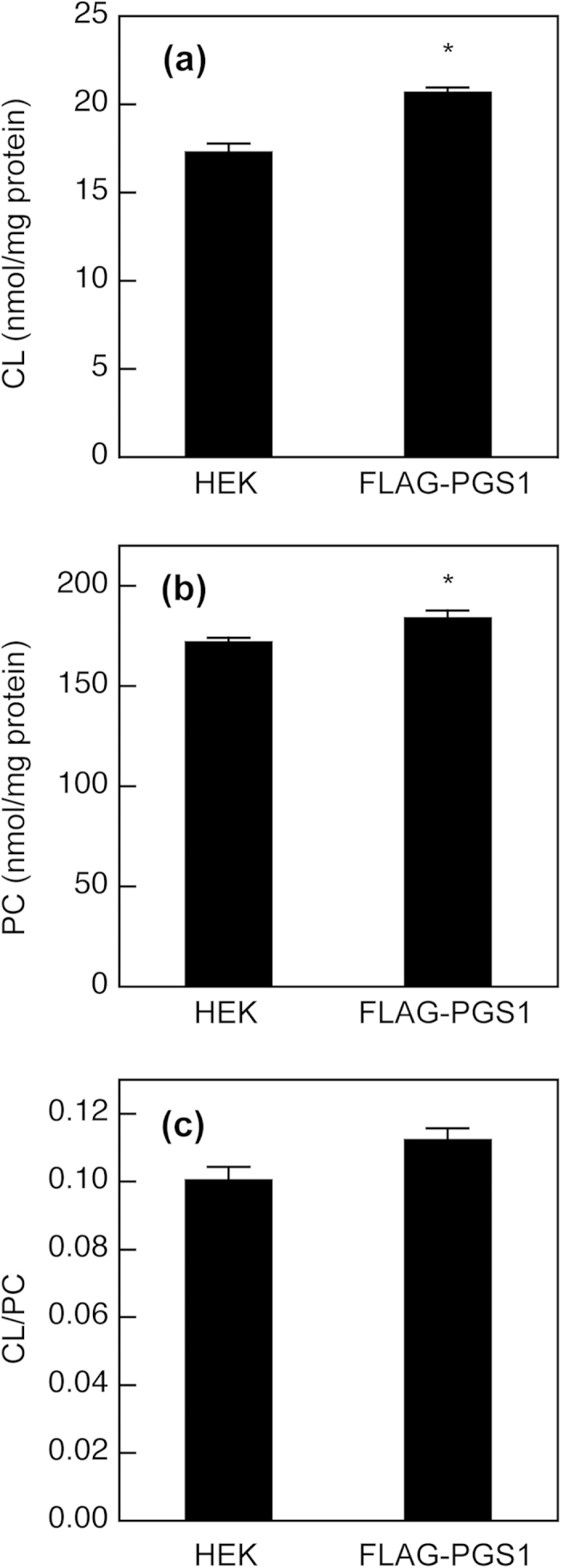
Effect of PGS1 overexpression on mitochondrial phospholipid composition. HEK293 and HEK/FLAG-PGS1 cells were seeded in 10-cm dishes at a density of 1.5 × 10^7^ cells and incubated with MEM supplemented with 10% FBS for 48 h at 37 °C. Then, the cells were incubated in MEM containing 0.02% BSA for 18 h at 37 °C. After incubation, purified mitochondrial fractions were isolated from the cells. CL content (**a**), PC content (**b**) and CL/PC ratio (**c**) of the purified mitochondrial fractions were determined by enzymatic measurements of CL and PC, and protein assay. Each bar represents the mean ± S.E. of three measurements. *P < 0.05, significantly different from HEK293 cells.

We next determined whether PGS1 overexpression affected the phospholipid composition in mitochondria. In mitochondria isolated from mammalian cells, the content of PG is >100-fold lower than that of CL^5^, and thereby not taken into account for the enzymatic measurement. As shown in Fig. 6a, the purified mitochondrial fraction from HEK/FLAG-PGS1 cells contained 1.2-fold more CL than that from HEK293 cells. On the other hand, FLAG-PGS1 expression also led to an increase in the PC content of the purified mitochondrial fraction (Fig. 6b), and induced no significant change in the mitochondrial CL/PC ratio (Fig. 6c). Although mitochondria cannot generate PC, PC is imported into mitochondria and is the most abundant phospholipid of mitochondrial membranes in mammalian cells^2^,^5^. Accordingly, the CL concentration in the mitochondrial membrane may be strictly maintained and not be altered by PGS1 overexpression.

**Figure 7:**
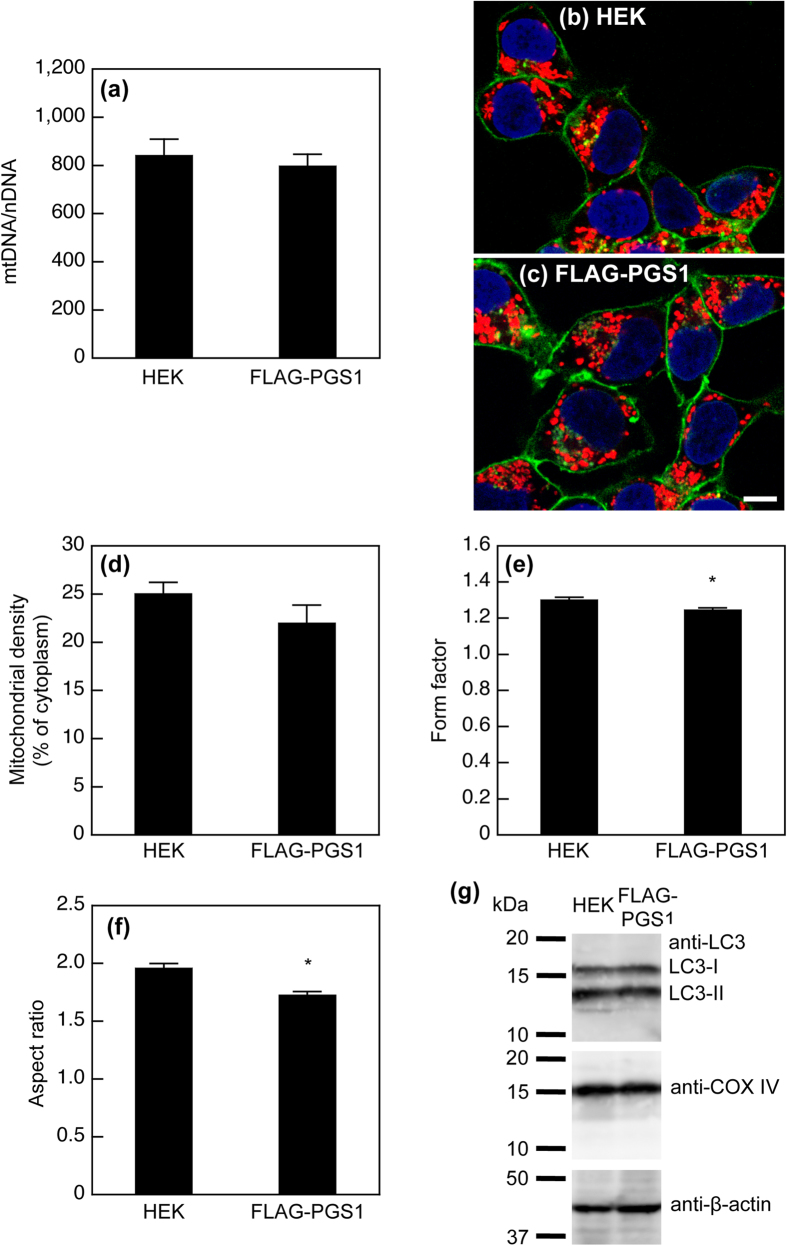
Effect of PGS1 overexpression on mitochondrial content and morphology. (**a**) Total DNA was extracted from the cells, and the nuclear gene *ASPOLG* and the mitochondrial gene *CCOI* were quantified by real-time PCR. The results are expressed as the ratio of the mitochondrial DNA copy number to the nuclear DNA copy number (mtDNA/nDNA). Each bar represents the mean ± S.E. of six measurements. There was no statistically significant difference between HEK293 and HEK/FLAG-PGS1 cells. (**b**) and (**c**) Confocal fluorescence microscopy of HEK293 and HEK/FLAG-PGS1 cells. Mitochondria, nuclei, and cell surfaces were visualized with MitoTracker Red CMXRos (red), DAPI (blue), and FITC-concanavalin A (green), respectively. The bar represents 20 μm. Confocal images were used to determine mitochondrial densities, form factors and aspect ratios. (**d**) Mitochondrial density is expressed as the percentage of cytoplasmic area occupied by mitochondria. Each bar represents the mean ± S.E. from 18 cells. (**e**) and (**f**) The form factor (perimeter^2^/4π·area) and the aspect ratio (major axis/minor axis) were calculated for each mitochondrial object (n = 427, HEK293; n = 410, HEK/FLAG-PGS1) in 18 cells per group. Both parameters have a minimal value of 1, which represents a perfect circle, and the values increase as the shape becomes elongate. Each bar represents the mean ± S.E. *P < 0.05, significantly different from HEK293 cells. (**g**) Immunoblot analysis of LC3. Sonically disrupted cell lysates (37.0 μg of protein) from HEK293 and HEK/FLAG-PGS1 cells were separated by 15% SDS-PAGE. LC3, COX IV, and β-actin were detected with specific antibodies.

To clarify whether the overexpression of PGS1 affects the number of mitochondria per cell or the amount of membrane per mitochondrion, we also assessed the ratio of the mitochondrial DNA copy number to the nuclear DNA copy number (mtDNA/nDNA) in HEK293 and HEK/FLAG-PGS1 cells by quantitative PCR. There was no significant difference in the mtDNA/nDNA ratio between HEK293 and HEK/FLAG-PGS1 cells (Fig. 7a), indicating that PGS1 overexpression had no effect on the number of mitochondria per cell. We consider that the overexpression of PGS1 simultaneously increased the CL content and the amount of mitochondrial membrane by enhancing the PC production and PC import into mitochondria.

Furthermore, by using confocal fluorescence microscopy, we analyzed the effect of PGS1 overexpression on the mitochondrial density and morphology. To visualize mitochondria, we stained HEK293 and HEK/FLAG-PGS1 cells with MitoTracker Red CMXRos, a widely used mitochondria-specific fluorophore (Fig. 7b,c). The cell surfaces and nuclei were visualized with fluorescein isothiocyanate (FITC)-concanavalin A and 4’,6-diamidino-2-phenylindole (DAPI), respectively. When impermeable cells are incubated with fluorescently labeled concanavalin A, its fluorescence is visible around the cells because the lectin binds only to plasma membrane glycoproteins^52^. As shown in Fig. 7d, the mitochondrial density in cytoplasm was similar in HEK293 and HEK/FLAG-PGS1 cells. For morphological analysis, we determined two parameters, form factor and aspect ratio^53^,^54^,^55^. The form factor is defined as the reciprocal value of the circularity. The aspect ratio of mitochondria is the ratio between the major and minor axes of an ellipse equivalent to the shape of mitochondria. Both parameters have a minimal value of 1 corresponding to a perfect circle, and the values increase as mitochondria elongate. The expression of FLAG-PGS1 caused slight reductions in the form factor and the aspect ratio of mitochondria (Fig. 7e,f), suggesting the transition from elongated to round morphology.

Damaged and unwanted mitochondria are selectively removed through mitophagy, which is a specific and selective form of autophagy. Mitophagy can be monitored through the degradation of mitochondrial proteins together with the appearance of autophagy markers such as microtubule-associated protein 1 light chain 3 (LC3)-II^56^. LC3-I is modified into the phosphatidylethanolamine-conjugated form, LC3-II. The conversion of LC3-I to LC3-II increases under stress conditions. COX IV is localized to the inner mitochondrial membrane. The expression level of COX IV and the ratio of LC3-II to LC3-I were not changed in HEK/FLAG-PGS1 cells (Fig. 7g). Thus, PGS1 overexpression may not induce mitophagy.

In conclusion, we developed and validated a novel enzymatic fluorometric assay for measuring PG + CL, which does not distinguish the species of fatty acyl chain. This simple method has high sensitivity and high accuracy, and allows many samples to be processed in parallel. In addition, all enzymes and substrates are available commercially. The usefulness of this procedure was demonstrated using cultured cells and purified mitochondria overexpressing PGS1. We demonstrated that the PG + CL content at low cell density was larger than that at high cell density, and that PGS1 overexpression led to the increased PG + CL contents in HEK293 cells and in purified mitochondria. Furthermore, this enzymatic fluorometric method may be applicable for measuring PG + CL under conditions where radiolabeling is difficult, such as in animal tissues and fluids. Therefore, this assay will be helpful to study the biological functions of PG and CL and their related cellular processes and disorders. High-throughput enzymatic methods will be developed for the measurements of all phospholipid classes in the near future.

## Methods

### Materials

PLD from *Streptomyces chromofuscus* was purchased from Asahi Kasei Pharma (Tokyo, Japan). GK from *Cellulomonas* sp. and GPO from *Pediococcus* sp. were obtained from Toyobo (Osaka, Japan). Peroxidase from horseradish roots was obtained from Oriental Yeast (Tokyo, Japan). Amplex Red reagent was purchased from Molecular Probes (Eugene, OR, USA). CL sodium salt from bovine heart, TOCL sodium salt, POPG sodium salt, PG sodium salt from chicken egg, PG sodium salt from soy, and L-α-monooleoyl phosphatidylglycerol were purchased from Avanti Polar Lipids (Alabaster, AL, USA). All other chemicals used were of the highest reagent grade.

### Enzymatic measurement of PG + CL

Measurement was performed using a three-reagent system. Reagent L1 contained 5 units/ml PLD, 1.5 mM CaCl_2_, 50 mM NaCl and 50 mM Tris-HCl (pH 7.4). Reagent L2 contained 5 units/ml GK, 5 units/ml GPO, 5 units/ml peroxidase, 300 μM Amplex Red, 0.2% Triton X-100, 4.5 mM ATP, 1.5 mM MgCl_2_, 50 mM NaCl and 50 mM Tris-HCl (pH 7.4). Amplex Red Stop Reagent was obtained from Molecular Probes. PG and CL standard solutions were dissolved in 1% Triton X-100 aqueous solution.

Sample (10 μl) was added to Reagent L1 (40 μl) and incubated at 37 °C for 30 min. After the incubation, Reagent L2 (50 μl) was added. After 30 min of incubation at room temperature, Amplex Red Stop Reagent (20 μl) was added. The fluorescence intensity (excitation 544 nm, emission 590 nm) was measured using a multimode microplate reader (Infinite M200, Tecan, Männedorf, Switzerland).

### Recombinant plasmid construction

Using PCR, an oligonucleotide encoding a FLAG (DYKDDDDK)-tagged epitope was appended to the 5’ end of the human PGS1 gene (GenBank: NM_024419). These PCR products were ligated into the Nhe I and Eco RI sites of the pIRESneo3 mammalian expression vector (Clontech, Mountain View, CA, USA) to generate the plasmids, pIRESneo3/FLAG-PGS1. pIRESneo3 contains the internal ribosome entry site, which permits the translation of two open reading frames from one messenger RNA. This expression system facilitates the establishment of pools of stably transfected cell lines whereby nearly all cells surviving in selective media express the gene of interest, since the neomycin phosphotransferase gene is expressed under the control of the same promoter^57^.

### Cell culture

HEK293 cells were grown in Dulbecco’s modified Eagle’s medium supplemented with 10% heat-inactivated fetal bovine serum (FBS) in a humidified incubator (5% CO_2_) at 37°C.

### Establishment of stable transformants of FLAG-PGS1

HEK293 cells were transfected with pIRESneo3/FLAG-PGS1 or pIRESneo3 (mock) using Lipofectamine Reagent and PLUS Reagent (Invitrogen, Carlsbad, CA, USA) according to the manufacturer’s instructions. Cells were selected with 1.2 mg/ml G418 disulfate, and a large number of G418-resistant clones were pooled in one dish. The expression of PGS1 was examined by Western blotting.

Total RNA isolation was performed using an SV Total RNA Isolation System (Promega, Madison, WI, USA). RT-PCR for *PGS1* mRNA was performed using an Access Quick RT-PCR system (Promega) with a set of primers (5’-CATCCCTCTACCTGGGGACA-3’ and 5’-CACCAGCTCCGTGAAGAAGT-3’). Another primer pair (5’-TGAACGGGAAGCTCACTGG-3’ and 5’-TCCACCACCCTGTTGCTGTA-3’) was used for the detection of *glyceraldehyde-3-phosphate dehydrogenase* (*GAPDH*) mRNA. The amplified product was electrophoresed on 3% (w/v) agarose gel with ethidium bromide.

For assessment of cell viability and intracellular ATP level, the cells were subcultured in 12-well plates at a density of 4.0 × 10^4^ cells in minimum essential medium (MEM) supplemented with 10% FBS. After incubation for 48 h, cell viability and intracellular ATP level were determined by the resazurin assay using a CellTiter-Blue cell viability assay kit (Promega) and by the luciferase-based luminescence assay using a CellTiter-Glo 2.0 assay kit (Promega), respectively.

### Western blotting

To prepare a whole cell lysate, cells were sonicated and lysed with PBS containing protease inhibitors (100 μg/ml (p-amidinophenyl)methanesulfonyl fluoride, 10 μg/ml leupeptin and 2 μg/ml aprotinin) and 1% Triton X-100. Purified mitochondrial, microsomal and cytosolic fractions were isolated from three dishes of cells according to the method previously described^58^. Samples were separated by SDS-PAGE on a 10% or 15% polyacrylamide gel calibrated with Precision Plus Protein WesternC Standards (Bio-Rad Laboratories, Hercules, CA, USA), transferred to PVDF membranes and immunoblotted with the monoclonal anti-FLAG antibody M2 (Sigma-Aldrich, St. Louis, MO, USA) (1:500 dilution), mouse polyclonal anti-PGS1 antibody (immunogen aa 110–556, Abnova, Taipei, Taiwan) (1:250 dilution), monoclonal anti-β-actin antibody AC-15 (Sigma-Aldrich) (1:1,000 dilution), rabbit monoclonal anti-cytochrome *c* oxidase subunit IV (COX IV) antibody 3E11 (Cell Signaling Technology, Danvers, MA, USA) (1:1,000 dilution) or rabbit polyclonal anti-LC3 antibody (Medical and Biological Laboratories, Nagoya, Japan) (1:1,000 dilution), and subsequently with horseradish peroxidase-conjugated goat anti-mouse IgG (Invitrogen) (1:4,000 dilution) or goat anti-rabbit IgG (Merck Millipore, Billerica, MA, USA) (1:10,000 dilution). Protein-antibody complexes were detected using ECL Plus Western blotting detection reagents and a biomolecular imager ImageQuant LAS 4000 mini (GE Healthcare, Buckinghamshire, UK).

### Measurement of PG + CL content in cells and purified mitochondria

For measurements of cellular phospholipid contents, the cells were subcultured in 10-cm dishes at various cell densities in MEM supplemented with 10% FBS. After incubation for 48 h, the cells were washed with fresh medium and incubated with MEM containing 0.02% bovine serum albumin (BSA) for 18 h at 37 °C. After incubation, the cells were chilled on ice, washed and scraped with cold PBS. The harvested cells were sonicated (model UR-20P, Tomy Seiko, Tokyo, Japan).

For measurements of phospholipid contents in purified mitochondrial fractions, the cells were subcultured in 10-cm dishes at a density of 1.5 × 10^7^ cells in MEM supplemented with 10% FBS. After incubation for 48 h, the cells were washed with fresh medium and incubated with MEM containing 0.02% BSA for 18 h at 37 °C. After incubation, the cells were chilled on ice, washed and scraped with cold phosphate-buffered saline (PBS). Purified mitochondrial fractions were isolated from the cells according to the method previously described^58^.

The protein concentrations in cell homogenates and purified mitochondrial fractions were measured using a BCA protein assay kit (Thermo Scientific, Rockford, IL, USA). Cellular or mitochondrial phospholipids were extracted by the method of Bligh and Dyer^59^, and dissolved in 1% Triton X-100. The contents of PC, PA and PG + CL in the lipid extract were measured by enzymatic assays^29^,^30^.

### Quantification of mitochondrial DNA

Cells were subcultured in six-well plates at a density of 2.5 × 10^6^ cells in MEM supplemented with 10% FBS. After incubation for 48 h, the cells were washed with fresh medium and incubated with MEM containing 0.02% BSA for 18 h at 37 °C. Total DNA was extracted from the cells using the QIAamp DNA Blood Mini kit (Qiagen, Hilden, Germany). For each DNA extract, the nuclear gene for human polymerase γ accessory subunit (ASPOLG) and the mitochondrial gene for human cytochrome *c* oxidase subunit I (CCOI) were quantified by real-time PCR with the use of a LightCycler480 Probe Master and a LightCycler 480 system (Roche Diagnostics, Mannheim, Germany), as described previously^60^. The results from the quantitative PCR are expressed as the ratio of the mean mitochondrial DNA copy number of duplicate measurements to the mean nuclear DNA copy number of duplicate measurements for a given extract (mtDNA/nDNA).

### Confocal fluorescence microscopy

Cells cultured on glass cover slips were incubated with 250 nM MitoTracker Red CMXRos (Molecular Probes) for 45 min at 37 °C. Then, the cells were fixed with 4% paraformaldehyde for 30 min. The plasma membrane glycoproteins and nuclei were stained with 20 μg/ml FITC-conjugated concanavalin A (Sigma-Aldrich) and 300 nM 4’, 6-diamidino-2-phenylindole (Sigma-Aldrich) for 5 min, respectively. Cover slips were mounted onto slides with SlowFade Diamond antifade mountant (Molecular Probes). The cells were viewed with a 100×/1.35 UPlanApo Oil Iris objective using a confocal microscope (FV1000-D, Olympus, Tokyo, Japan). Quantitative analyses of mitochondrial morphology and density in cytoplasm were performed using ImageJ software (National Institutes of Health). Mitochondrial density is determined as the percentage of cytoplasmic area occupied by mitochondria. The form factor (perimeter^2^/4π·area) and the aspect ratio (major axis/minor axis) were calculated for each mitochondrial object^53^,^54^,^55^.

### Statistical analysis

The statistical significance of differences between mean values was analyzed using the non-paired t-test. Multiple comparisons were performed using the Bonferroni test following ANOVA. Differences were considered significant at *P* < 0.05. Unless indicated otherwise, results are given as mean ± S.E.

## Additional Information

**How to cite this article**: Morita, S.-y and Terada, T. Enzymatic measurement of phosphatidylglycerol and cardiolipin in cultured cells and mitochondria. *Sci. Rep.* **5**, 11737; doi: 10.1038/srep11737 (2015).

## Supplementary Material

Supplementary Information

## Figures and Tables

**Table 1:** Recovery of CL added to the cellular lipid extract.

Added (μM)	Measured (μM)	Expected (μM)	Recovery (%)
0	36.4		
12.5	48.9	48.9	99.9
25.0	61.3	61.4	99.7
50.0	87.7	86.4	101.5
75.0	110.0	111.4	98.7
100.0	138.1	136.4	101.2

The heart CL standard solution was added to the lipid extract from HEK293 cells. The concentrations of PG + CL were measured by the enzymatic assay.
