# Supplementary material for: Enzymatic measurement of phosphatidylglycerol and cardiolipin in cultured cells and mitochondria
Source: Sci Rep. 2015 Jun 30;5:11737. doi: 10.1038/srep11737 (PMC4485230; doi:10.1038/srep11737)
Supplement: Supplementary Information [file srep11737-s1.pdf]

Supplementary Information

Enzymatic measurement of phosphatidylglycerol and cardiolipin in cultured cells and mitochondria

Shin-ya Morita* and Tomohiro Terada

Department of Pharmacy, Shiga University of Medical Science Hospital, Otsu City, Shiga 520-2192, Japan

Supplementary Methods

Materials

H_2O_2 was purchased from Wako Pure Chemical Industries. *sn*-Glycerol-3-phosphate (G3P) bis(cyclohexylammonium) salt was obtained from Sigma-Aldrich. G3P oxidase (GPO) from *Pediococcus* sp. was obtained from Toyobo. Peroxidase from horseradish roots was obtained from Oriental Yeast. Amplex Red and Amplex Red Stop Reagent were purchased from Molecular Probes. All other chemicals used were of the highest reagent grade.

Removal of H_2O_2 and G3P by lipid extraction

H_2O_2 and G3P were solubilized in water. The lipid extraction by the Bligh and Dyer method [1] was performed on the solutions without and with H_2O_2 (10, 100 and 1000 μM) or G3P (10, 50, 100 and 500 μM). In brief, 3.75 ml of chloroform/methanol (1:2) was added to 1 ml of the sample solution and vortexed. Then, 1.25 ml of chloroform was added to the sample and vortexed. Then, 1.25 ml of water was added in the tube. After vortexing, the sample stood overnight at 4°C. The phase split was completed by centrifugation. The upper aqueous phase and the interfacial material were removed carefully. The recovered lower organic phase was washed with the blank upper phase solution, which is the upper phase of the mixed solution consisting of chloroform (2.5 ml), methanol (2.5ml) and water (2.25 ml). The upper phase was removed again, and the organic solvent of the lower phase was evaporated. The evaporated sample was dissolved in 1% Triton X-100 (200 μl).

H_2O_2 measurement

The enzymatic assay has been widely used to determine the concentration of H_2O_2 [2,3]. The reaction buffer contained 50 mM NaCl and 50 mM Tris-HCl (pH 7.4). Reagent HP contained 5 units/ml peroxidase, 300 μM Amplex Red, 0.2% Triton X-100, 50 mM NaCl and 50 mM Tris-HCl (pH 7.4). The reaction buffer (40 μl) and Reagent HP (50 μl) were added to the sample (10 μl). After 30 min of incubation at room temperature, Amplex Red Stop

Reagent (20 μl) was added. The fluorescence intensity (excitation 544 nm, emission 590 nm) was measured using a multimode microplate reader (Infinite M200, Tecan).

G3P measurement

The enzymatic assay has been widely used to determine the concentration of G3P [4]. The reaction buffer contained 50 mM NaCl and 50 mM Tris-HCl (pH 7.4). Reagent GP contained 5 units/ml GPO, 5 units/ml peroxidase, 300 μM Amplex Red, 0.2% Triton X-100, 50 mM NaCl and 50 mM Tris-HCl (pH 7.4). The reaction buffer (40 μl) and Reagent HP (50 μl) were added to the sample (10 μl). After 30 min of incubation at room temperature, Amplex Red Stop Reagent (20 μl) was added. The fluorescence intensity (excitation 544 nm, emission 590 nm) was measured using a multimode microplate reader (Infinite M200).

References

1. Bligh, E.G. & Dyer, W.J. A rapid method of total lipid extraction and purification. *Can. J. Biochem. Physiol.* **37**, 911-917 (1959).
2. Zhou, M., Diwu, Z., Panchuk-Voloshina, N. & Haugland, R.P. A stable nonfluorescent derivative of resorufin for the fluorometric determination of trace hydrogen peroxide: applications in detecting the activity of phagocyte NADPH oxidase and other oxidases. *Anal. Biochem.* **253**, 162-168 (1997).
3. Mohanty, J.G., Jaffe, J.S., Schulman, E.S. & Raible, D.G. A highly sensitive fluorescent micro-assay of H2O2 release from activated human leukocytes using a dihydroxyphenoxazine derivative. *J. Immunol. Methods* **202**, 133-141 (1997).
4. Skelly, R.H., Bollheimer, L.C., Wicksteed, B.L., Corkey, B.E. & Rhodes, C.J. A distinct difference in the metabolic stimulus-response coupling pathways for regulating proinsulin biosynthesis and insulin secretion that lies at the level of a requirement for fatty acyl moieties. *Biochem. J.* **331 (Pt 2)**, 553-561 (1998).

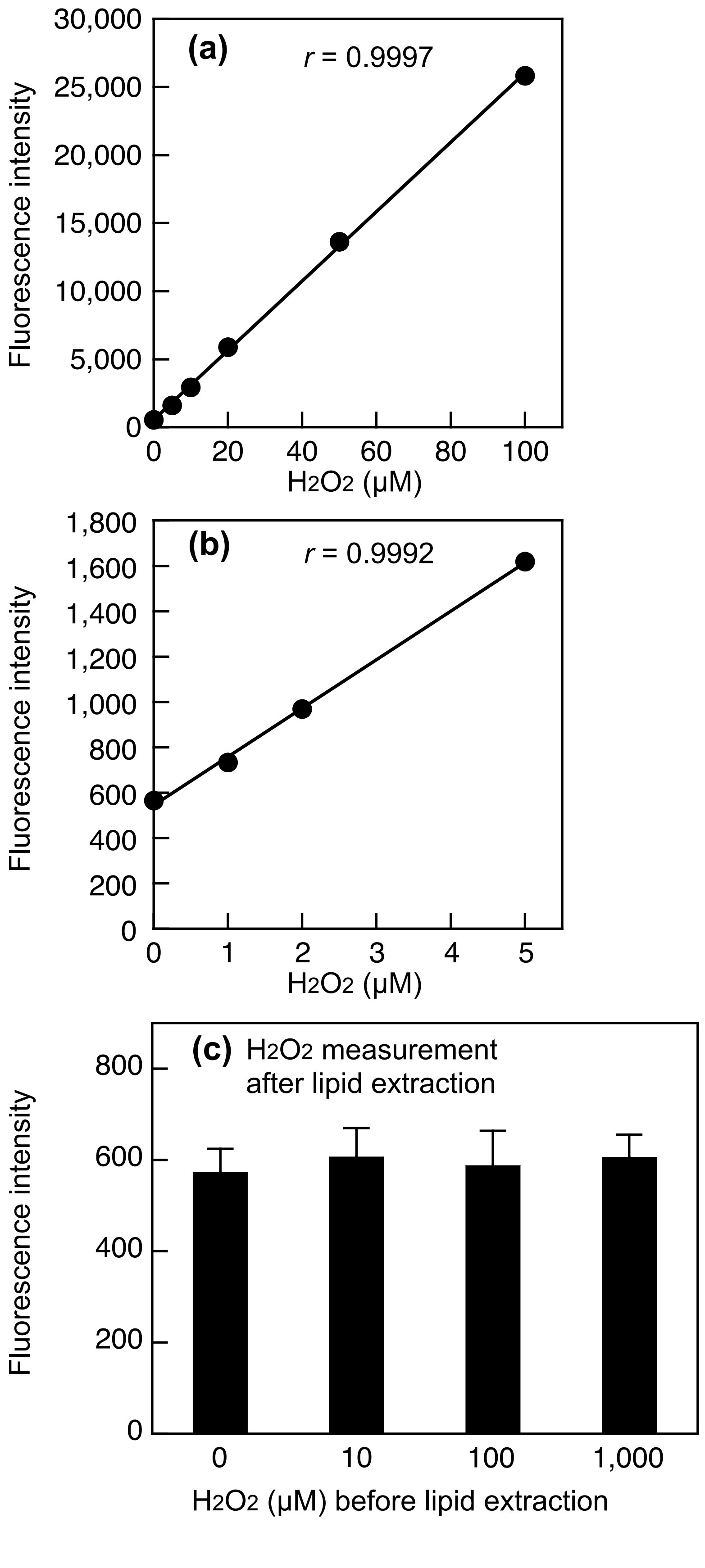

Supplementary Figure S1. Removal of H_2O_2 by lipid extraction. (a) and (b) Standard curves for H_2O_2 measurement. Background fluorescence was 566. Each point represents the mean of duplicate measurement. The correlation coefficients were r = 0.9997 (a) and r = 0.9992 (b). (c) Fluorescence changes in response to the H_2O_2 solutions after the lipid extraction by the method of Bligh and Dyer. Before the lipid extraction, the solutions contained the indicated concentrations of H_2O_2. In H_2O_2 measurement after the lipid extraction, there were no statistically significant differences in the fluorescence intensities among these samples.

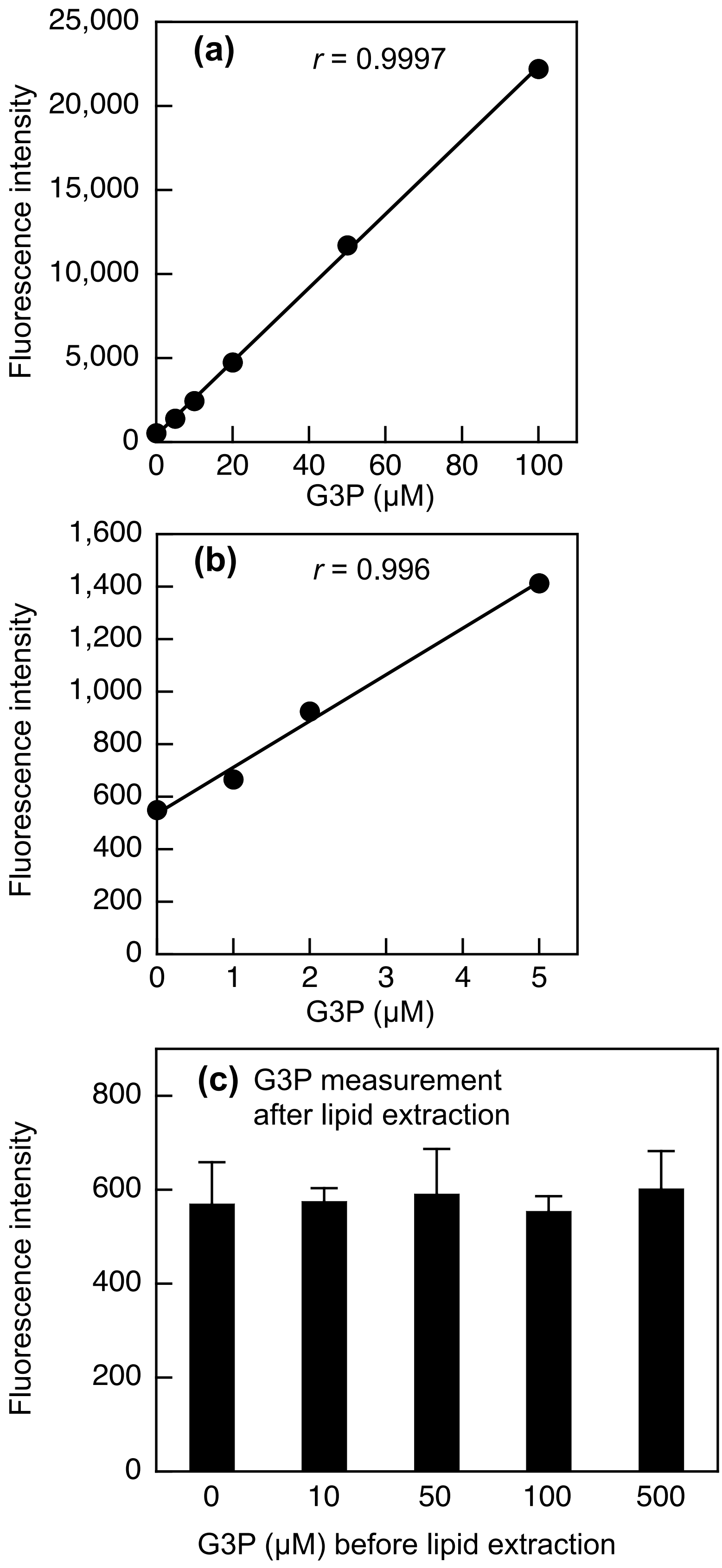

Supplementary Figure S2. Removal of G3P by lipid extraction. (a) and (b) Standard curves for G3P measurement. Background fluorescence was 551. Each point represents the mean of duplicate measurement. The correlation coefficients were $r = 0.9997$ (a) and $r = 0.996$ (b). (c) Fluorescence changes in response to the G3P solutions after the lipid extraction by the method of Bligh and Dyer. Before the lipid extraction, the solutions contained the indicated concentrations of G3P. In G3P measurement after the lipid extraction, there were no statistically significant differences in the fluorescence intensities among these samples.

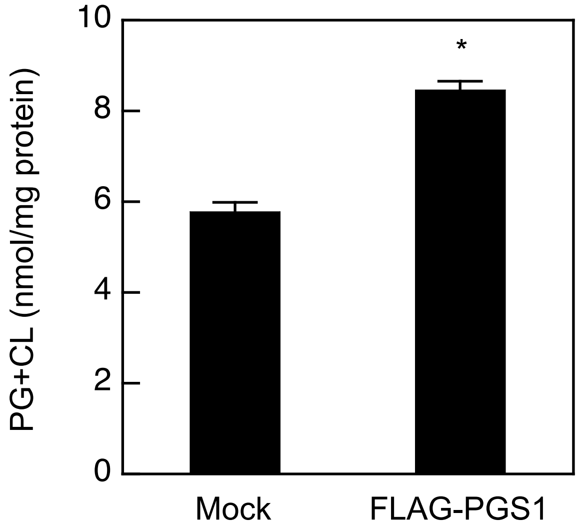

Supplementary Figure S3. PG+CL contents of mock-transfected HEK293 and HEK/FLAG-PGS1 cells. HEK293 cells stably transfected with the pIRESneo3 vector (Mock) and HEK/FLAG-PGS1 cells on 10 cm dishes were incubated in MEM containing 0.02% BSA for 18 h at 37°C. There was no difference in the densities of mock and HEK/FLAG-PGS1 cells (79.9 ± 1.0 and 75.0 ± 1.9 μg protein/cm^2, respectively). PG+CL content of the cells was determined by the enzymatic measurement of PG+CL and protein assay. Each bar represents the mean ± S.E. of three measurements. *P<0.05, significantly different from the mock cells.
